# Developing an understanding of artificial intelligence lung nodule risk prediction using insights from the Brock model

**DOI:** 10.1007/s00330-022-08635-4

**Published:** 2022-03-03

**Authors:** Madhurima R. Chetan, Nicholas Dowson, Noah Waterfield Price, Sarim Ather, Angus Nicolson, Fergus V. Gleeson

**Affiliations:** 1 Department of Radiology, John Radcliffe Hospital, Oxford University Hospitals NHS Foundation Trust, X-ray Level 2, Headley Way, Headington, Oxford, OX3 9DU UK; 2 Nuffield Department of Surgical Sciences, University of Oxford, John Radcliffe Hospital, Headley Way, Headington, Oxford, OX3 9DU UK; 3 Optellum Ltd, Oxford Centre for Innovation, Oxford, OX1 1BY UK; 4 Department of Oncology, University of Oxford, Old Road Campus Research Building, Roosevelt Drive, Oxford, OX3 7DQ UK

**Keywords:** Artificial intelligence, Neural networks, Computer, Algorithms, Early detection of cancer, Multidetector computed tomography

## Abstract

**Objectives:**

To determine if predictions of the Lung Cancer Prediction convolutional neural network (LCP-CNN) artificial intelligence (AI) model are analogous to the Brock model.

**Methods:**

In total, 10,485 lung nodules in 4660 participants from the National Lung Screening Trial (NLST) were analysed. Both manual and automated nodule measurements were inputted into the Brock model, and this was compared to LCP-CNN. The performance of an experimental AI model was tested after ablating imaging features in a manner analogous to removing predictors from the Brock model. First, the nodule was ablated leaving lung parenchyma only. Second, a sphere of the same size as the nodule was implanted in the parenchyma. Third, internal texture of both nodule and parenchyma was ablated.

**Results:**

Automated axial diameter (AUC 0.883) and automated equivalent spherical diameter (AUC 0.896) significantly improved the accuracy of Brock when compared to manual measurement (AUC 0.873), although not to the level of the LCP-CNN (AUC 0.936). Ablating nodule and parenchyma texture (AUC 0.915) led to a small drop in AI predictive accuracy, as did implanting a sphere of the same size as the nodule (AUC 0.889). Ablating the nodule leaving parenchyma only led to a large drop in AI performance (AUC 0.717).

**Conclusions:**

Feature ablation is a feasible technique for understanding AI model predictions. Nodule size and morphology play the largest role in AI prediction, with nodule internal texture and background parenchyma playing a limited role. This is broadly analogous to the relative importance of morphological factors over clinical factors within the Brock model.

**Key Points:**

• *Brock lung cancer risk prediction accuracy was significantly improved using automated axial or equivalent spherical measurements of lung nodule diameter, when compared to manual measurements*.

• *Predictive accuracy was further improved by using the Lung Cancer Prediction convolutional neural network, an artificial intelligence-based model which obviates the requirement for nodule measurement*.

• *Nodule size and morphology are important factors in artificial intelligence lung cancer risk prediction, with nodule texture and background parenchyma contributing a small, but measurable, role*.

**Supplementary Information:**

The online version contains supplementary material available at 10.1007/s00330-022-08635-4.

## Introduction

Pulmonary nodules are a common incidental finding on computed tomography (CT) [1]. Current guidelines emphasise the importance of assessing the likelihood that a nodule is malignant, with further management being dependent on the predicted risk of malignancy [2, 3]. Lung cancer risk prediction models have been developed using both statistical approaches such as logistic regression (LR) and machine learning approaches such as convolutional neural networks (CNN).

The Brock University model is a LR model that has been successfully validated in a screening cohort from the National Lung Screening Trial (NLST) in the USA and in an unselected clinical population in the UK [4–8]. The British Thoracic Society (BTS) guidelines recommend the use of the Brock model in clinical practice, whilst the Fleischner Society guidelines do not advocate any risk prediction model but do acknowledge that the Brock model is of great interest [2, 3].

Nodule size, defined as the maximum diameter of the long axis of the nodule measured by a thoracic radiologist using electronic callipers, is the single most important predictor in the Brock model [8]. Other predictors of cancer in this model include older age, female sex, family history of lung cancer, emphysema, upper lobe nodule location, part-solid nodule, lower nodule count, and spiculation.

Manual nodule diameter measurements are subject to significant intra- and inter-reader variability, greatest at measurements of 5 mm and 6 mm, which are the key thresholds for determining follow-up [9, 10]. Moreover, diameter does not accurately reflect nodule size, unless nodules are perfectly spherical. Automated measurements of nodule diameter and volume have produced no or modest improvement in the predictive accuracy of the Brock model in the literature [11, 12].

Artificial intelligence (AI) models are a step forward from automated nodule measurement as they typically do not require nodule measurement or data entry. The Lung Cancer Prediction CNN (LCP-CNN) is an externally validated AI model, which has been shown to outperform the Brock model in the NLST cohort and a UK clinical cohort [13, 14]. However, whilst the Brock model is fully interpretable, the rationale underlying predictions made by LCP-CNN is not well understood and the effects of individual predictors cannot be isolated.

We hypothesise that predictions made by LCP-CNN are, in part, attributable to those imaging features which are also predictors in the Brock model. First, we propose that LCP-CNN does more than just measuring nodule size optimally. We compare the predictive accuracy of LCP-CNN against that of automated measurements within the Brock model. Second, we hypothesise that ablating imaging features is analogous to removing predictors from the Brock model. We explore which imaging features contribute to the predictions of LCP-CNN by re-training the CNN on information-ablated CT images and assessing the drop in performance attributable to each ablated feature.

## Materials and methods

### Study dataset

This is a retrospective analysis of imaging data from the NLST. Trial design and eligibility criteria are described elsewhere [15]. In brief, NLST was a multicentre randomised trial of three rounds of screening with low-dose CT compared to chest radiography for asymptomatic participants aged 55–74 years with a significant smoking history. Participants were followed for lung cancer diagnoses for a median of 6.5 years. Nodule size was measured using electronic callipers by NLST radiologists who had received training in standardised image interpretation. No standard protocol for nodule evaluation was mandated.

**Fig. 1:**
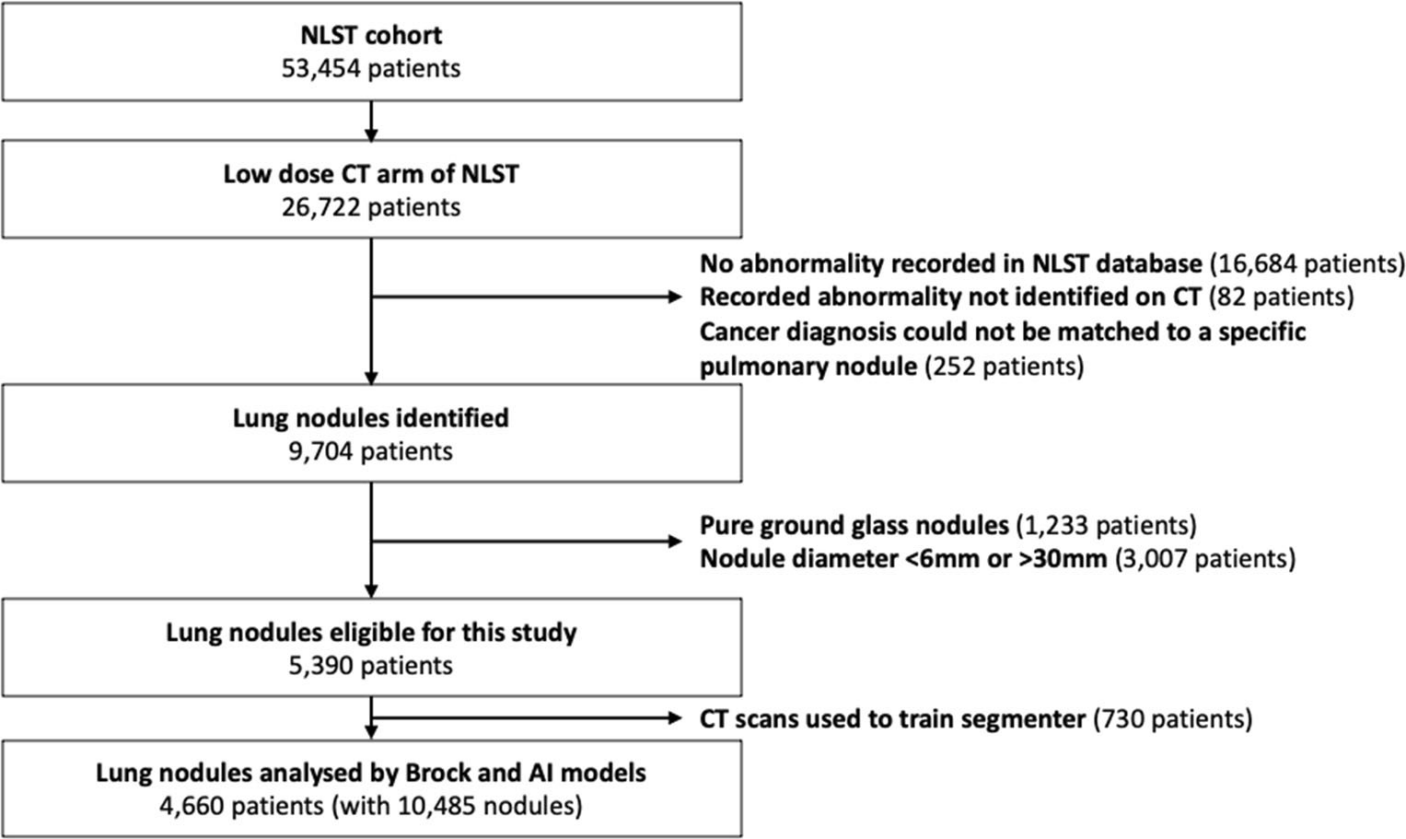
Study flow diagram

Of the 26,722 patients in the CT screening arm of the NLST, 16,684 were excluded as no abnormality was recorded in the NLST database. In our study, CT studies from 10,038 patients with recorded abnormalities were reviewed under the supervision of an experienced radiologist to identify pulmonary nodules. Each nodule was manually annotated and its correspondence to an abnormality found during NLST recorded. All time-points were considered and nodules were tracked over time. Eighty-two patients had no recorded abnormalities that could be matched to a CT finding. Two hundred fifty-two patients with a diagnosis of cancer were excluded because their cancer diagnosis could not be matched to a specific nodule. Nodules that were not solid or part-solid were excluded (*n* = 1233 patients) because the LCP-CNN was trained on solid and part-solid nodules only. Nodules < 6 mm or > 30 mm using manual measurements were excluded (*n* = 3007 patients). Nodules < 6 mm were excluded because these do not routinely warrant surveillance according to the Fleischner Society, and masses > 30 mm were excluded because the online Brock calculator and segmentation algorithm were not designed for masses > 30 mm [3, 16, 17]. In total, 4660 participants with 10,485 nodules were included in the analysis. The study flow diagram is provided in Fig. 1.

### Automatic nodule size measurements

The U-Net convolutional neural network is a well-established medical segmentation tool that was adapted for nodule segmentation within this study [16, 18]. In total, 1276 participants were randomly selected to train the segmentation algorithm. Of these, participants meeting the inclusion criteria for the analysis in this study (*n* = 730) were excluded from the validation cohort.

Volumetric segmentation was initiated from a seed point within the nodule identified by doctors under the supervision of a senior chest radiologist (F.V.G.), and then performed by the algorithm in an unsupervised manner. Equivalent spherical diameter was calculated using ∛(6/π.*V*), where *V* is nodule volume. Two different methods were used to measure maximal axial diameter. In the first, the longest distance between any two points on the nodule boundary was calculated on each axial slice, and the maximum among all axial slices was used. However, this method can overestimate the diameter of spiculated nodules. In the second method, the largest diameter was calculated for an ellipse fitted to each axial contour using standard least squares methods. Both methods gave almost identical results. We have reported only the second because it is less sensitive to spiculation and small changes in nodule geometry, in line with Fleischner Society recommendations [19]. Some of these results have been previously published in the form of an abstract and conference proceeding [20, 21].

For each participant, the Brock model was used to calculate risk of malignancy using (1) manual diameter provided in the NLST dataset, (2) maximal axial diameter derived from automatic segmentation, and (3) equivalent spherical diameter derived from automatic segmentation. The risk of malignancy was also derived using the LCP-CNN. The LCP-CNN development and validation are fully described in prior publications, and the same version of the model was used in this analysis [13, 14].

Predictive accuracy was primarily evaluated with area under the receiver operating characteristic curve (AUC) analysis. The statistical significance of any difference in accuracy between the methods was computed from the distribution of AUC differences. This was derived by bootstrapping across 10,000 draws from the data with replacement. 95% confidence intervals (CI) were obtained from the distribution of differences. *p* values were computed using a two-sided permutation test using 10,000 random resamplings of the data [22], with *p* < 0.05 considered statistically significant.

### Information ablation

Covariates were removed from the full Brock model, and the predictive performance of three ‘feature-reduced’ Brock models was tested.

- Non-morphological factors only

Age, sex, emphysema, family history of cancer, nodule location, and nodule count were included.

- Morphological factors only

Nodule size, nodule type (solid or part-solid), and spiculation were included.

- Without spiculation (equivalent to the ‘parsimonious model’ in [3])

All covariates in the full Brock model, except spiculation, were included.

Unlike the Brock model, LCP-CNN does not consist of human-interpretable terms. Hence, feature removal was performed by ablating information from the CT images. As the LCP-CNN was not trained to analyse ablated CT images, an experimental AI model was trained to predict malignancy from ablated CT images using the dataset and same eight folds as were used to train the LCP-CNN model [13, 14]. For a given fold, three-quarters of the data was partitioned for training the AI model, one-eighth was partitioned for validation, and one-eighth was partitioned for testing. Each participant was assigned to be in the test partition in precisely one of each of the folds. Each fold had an approximately equal proportion of participants with malignant nodules. Each of the eight folds was associated with a single corresponding independently trained model. During analysis, the results of the eight folds were combined together to provide a set of cross-validation results for the entire dataset as described in prior publications [13, 14].

The predictive performance of the AI model was tested on unmodified and ablated CT images.

- Parenchyma only

All information about the nodule was ablated. A region 15 mm away from the furthermost edge of the nodule margin towards the hilum was evaluated, comprising of an image containing background lung parenchyma but without the nodule being visible.

- Morphological factors

All information about the background lung and the nodule internal texture was ablated. Background lung was replaced with average lung density across all patients (− 825 Hounsfield units), and nodule internal texture was replaced with mean nodule density.

- Implanted sphere

A sphere of the same volume as the nodule and with mean nodule density was implanted in the ‘parenchyma only’ model as described above.

As information ablation was carried out using mean nodule density, a subgroup analysis was performed to compare the predictive performance of the experimental AI model on part-solid and solid nodules.

### Data analysis

Data analysis was performed using Python 3.8 installed on Ubuntu 20.04 with NumPy 1.19.4, scikit-learn 0.21.3, and pandas 0.23.4 libraries.

## Results

In total, 4660 participants with 10,485 lung nodules (of which 556 were malignant) were included in this retrospective analysis. Demographic data are provided in Table 1.

**Table 1:** Study participant demographics and characteristics of pulmonary nodules

	All	Benign	Cancer
Participants (*n*, %)	4660 (100)	4224 (90.6)	436 (9.4)
Age (years) (mean, SD)	62.8 (5.2)	62.1 (5.2)	64.4 (5.4)
Female (*n*, %)	1815 (38.9)	1627 (38.5)	188 (43.1)
Current smoker at enrolment (*n*, %)	2323 (49.8)	2079 (49.2)	244 (56.0)
Smoking history (pack-years) (mean, SD)	58.1 (24.5)	57.3 (24.0)	65.7 (28.1)
Years since quitting smoking (mean, SD)	7.3 (4.7)	7.4 (4.7)	6.5 (4.4)
Emphysema (*n*, %)	4124 (50.1)	3805 (49.5)	319 (58.7)
Personal history of cancer (*n*, %)	197 (4.2)	167 (4.0)	30 (6.9)
Family history of cancer (*n*, %)	1052 (22.6)	946 (22.4)	106 (24.3)
Pulmonary nodules (*n*, %)	10,485 (100)	9929 (94.7)	556 (5.3)
Solitary nodule (*n*, %)	4491 (54.5)	4190 (54.5)	301 (55.4)
Upper lobe location (*n*, %)	3475 (33.1)	3134 (31.6)	341 (61.3)
Spiculation (*n*, %)	1433 (13.7)	1153 (11.6)	280 (50.4)
Part-solid nodule (*n*, %)	669 (6.4)	594 (6.0)	75 (13.5)

Abbreviations: *n*, number; *%*, percentage; *SD*, standard deviation

Malignant nodules were larger than benign nodules, regardless of measurement technique (Supplementary Figure 1). Equivalent spherical diameter was smaller than manual or automatic maximal axial diameter, as many nodules were not perfectly spherical.

**Fig. 2:**
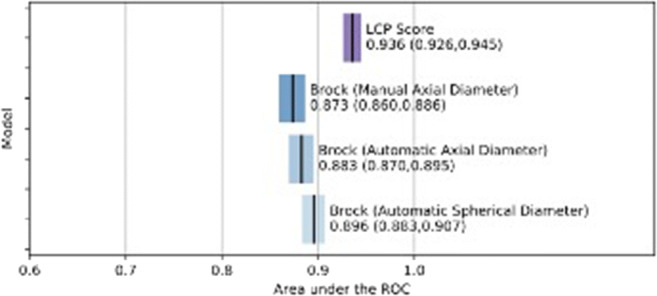
Area under the receiver operating characteristic curve (ROC) for the Brock model using manual and automatic measurement techniques and for Lung Cancer Prediction convolutional neural network (LCP-CNN)

AUC values for the Brock model were significantly higher with automatic axial diameter (0.883, 95% CI 0.870–0.895, *p* < 0.02) and automatic spherical diameter (0.896, 95% CI 0.883–0.907, *p* < 0.0001) than with manual diameter (0.873, 95% CI 0.860–0.886) (Fig. 2). Within the automatic techniques, equivalent spherical diameter had a significantly greater AUC than maximal axial diameter (*p* < 0.0001). LCP-CNN showed significantly greater AUC than Brock regardless of measurement technique (0.936, 95% CI 0.926–0.945, *p* < 0.0001) (Fig. 2).

**Fig. 3:**
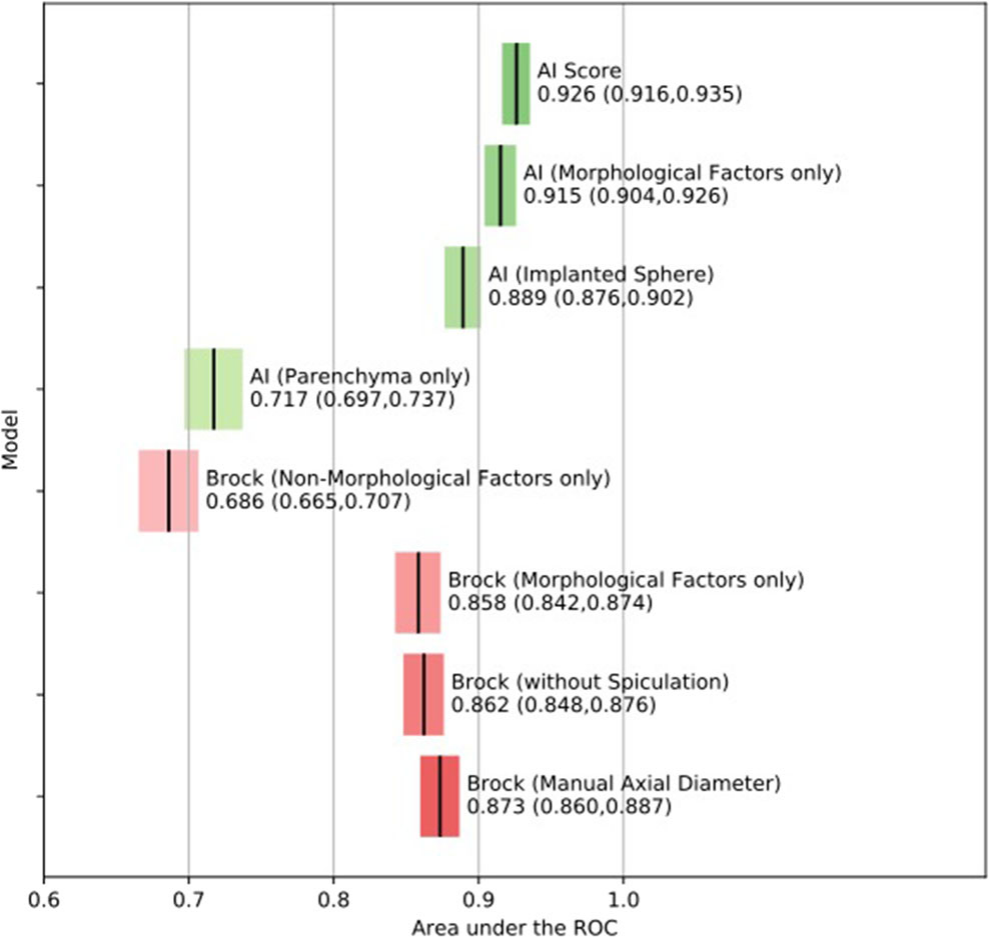
Area under the receiver operating characteristic curve (ROC) for feature-reduced Brock models and information-ablated artificial intelligence (AI) models

The predictive performance of the various feature-reduced Brock models is presented in Fig. 3 and Supplementary Figure 2. The Brock model with non-morphological factors only was a poor predictor of malignancy, with AUC 0.686 (95% CI 0.665–0.706). On the other hand, the Brock model with morphological factors only was a good predictor with AUC 0.858 (95% CI 0.842–0.874). The Brock model without spiculation performed slightly better with AUC 0.862 (95% CI 0.848–0.876, *p* 0.16) but the difference was not significant.

The predictive performance of the experimental AI model is presented in Fig. 3 and Supplementary Figure 3. The experimental AI model with parenchyma alone and no visible nodule produced a poor predictive performance with AUC 0.717 (95% CI 0.697–0.737). When a sphere of the same volume and mean density as the nodule was implanted into this parenchyma, the predictive performance was good with AUC 0.889 (95% CI 0.876–0.901). When the background lung parenchyma and the nodule internal texture were replaced with uniform density, predictive performance was very good with AUC 0.915 (95% CI 0.904–0.926), compared to AUC 0.926 (95% CI 0.916–0.935) for unablated images.

**Fig. 4:**
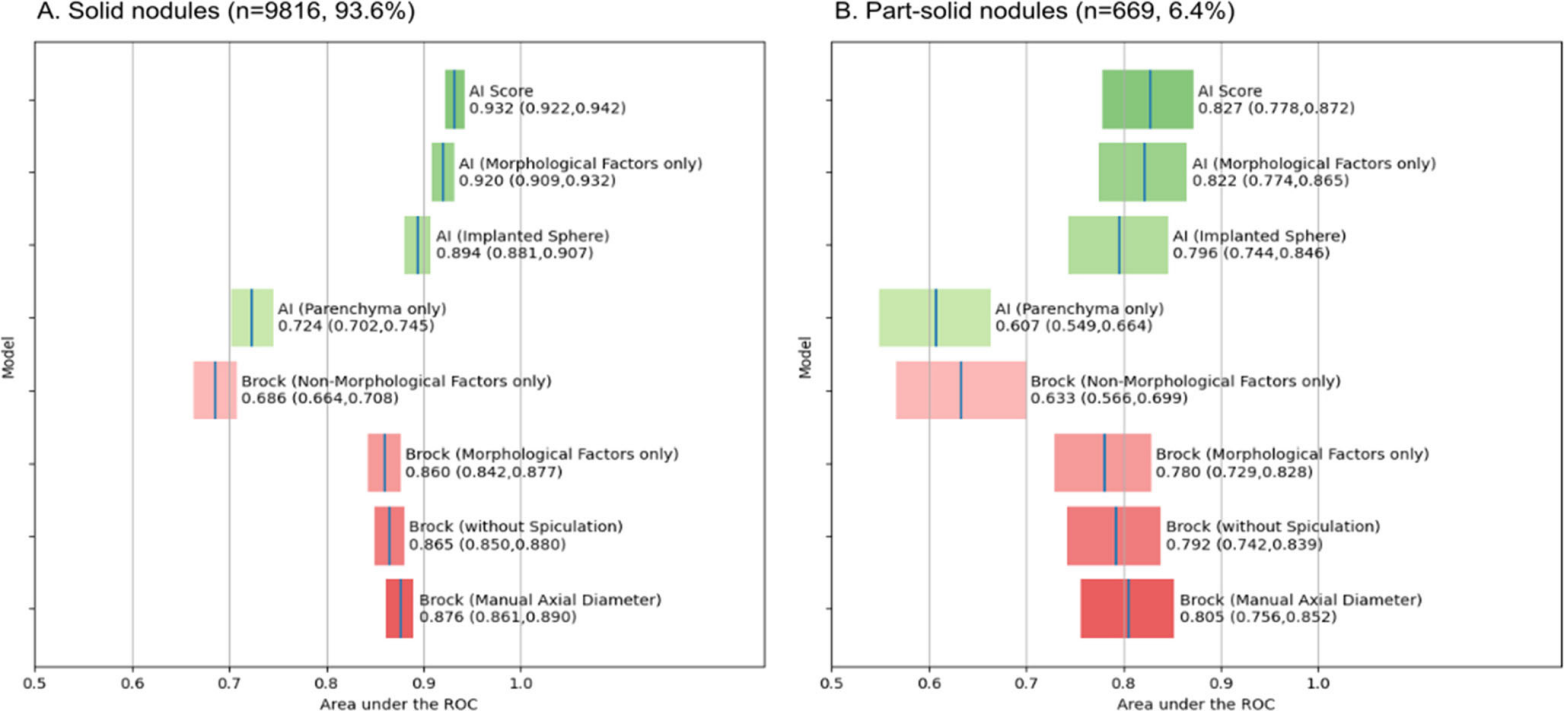
A subgroup analysis of solid nodules (**A**) and part-solid nodules (**B**) showing area under the receiver operating characteristic curve (ROC) for feature-reduced Brock models and information-ablated artificial intelligence (AI) models

A subgroup analysis was undertaken to compare predictive performance for part-solid nodules (*n* = 669, 6.4%) and solid nodules (*n* = 9816, 93.6%). The results are presented in Fig. 4. On unablated images, performance of the experimental AI model was reduced for part-solid nodules (AUC 0.827, 95% CI 0.778–0.872) compared to solid nodules (AUC 0.932, 95% CI 0.992–0.942). A similar reduction in performance was observed in the two ablation experiments where mean nodule density was employed (AUC 0.822 vs 0.920, and AUC 0.796 vs 0.894) and in the parenchyma-only ablation experiment (AUC 0.607 vs 0.724).

## Discussion

Automated measurement (AUC 0.883 and 0.896, *p =* 0.02 and *p* < 0.0001) significantly improved the accuracy of the Brock model compared with manual measurement (AUC 0.873). We report a larger effect size than prior studies which demonstrated that automated measurement is associated with no or modest improvement in the accuracy of the Brock model. This may be due to different methods used in our study; the Brock model was not re-fitted to the automated data [12] and the sample size was sixteen-fold greater [11]. Within the automated techniques, equivalent spherical diameter resulted in a significant increase in accuracy (AUC 0.896, *p* < 0.0001) compared to maximal axial diameter (AUC 0.883). This suggests that the volumetric nature of automatic measurement may underpin the improvement in predictive performance. The merits of nodule volume over diameter in risk prediction have been described elsewhere [23]. Equivalent spherical diameters offer an alternative to volume that remains compatible with the Brock model.

LCP-CNN significantly improved the accuracy of lung cancer prediction (AUC 0.936, *p* < 0.0001) when compared to the Brock model supplemented with automated measurement. This suggests that LCP-CNN does more than just measuring nodule size optimally. Our results are supported by another study showing that LCP-CNN outperformed the Brock model in the IDEAL cohort, a cohort of non-screen-detected incidental pulmonary nodules in the UK [13].

An experimental AI model was trained on ablated CT images in order to test the hypothesis that predictions made by LCP-CNN are, in part, attributable to those imaging features which are also predictors in the Brock model. When tested on unablated CT images, the experimental AI model (AUC 0.926) performed similarly to the LCP-CNN (AUC 0.936). The small performance gap here is likely due to some of the capacity of the experimental model being used to characterise features in the ablated CT images that differ from those in unmodified CT images.

Morphological factors within the Brock model, such as size, spiculation, and type, are all visible to the experimental AI model. Morphological features alone (AUC 0.858) were almost as good as the full Brock model in predicting malignancy (AUC 0.896), with non-morphological features playing a limited role. We hypothesised that, analogous to the Brock model, the experimental AI model predominantly uses information on nodule morphology to predict cancer, with parenchyma playing a limited role. Indeed, ablating all information on the nodule and leaving only the background parenchyma gave a poor performance (AUC 0.717)—but this is still better than a random classifier and better than Brock with non-morphological factors only (AUC 0.686). The AI is blind to the clinical data used by the Brock model; however, it may have learnt parenchymal changes indicative of age, emphysema, and environmental exposures, hence performing better than random [24].

Replacing the background parenchyma and the nodule with uniform density, whilst preserving the nodule morphology, produced a good predictive performance (AUC 0.915). In principle, this is analogous to Brock with morphological factors only (AUC 0.858). The difference in performance suggests that the AI utilises morphologic features in addition to those within Brock, and possibly outside of the radiology lexicon. This is supported by a study on size-matched benign and malignant nodules showing reasonable performance of LCP-CNN independent of size [25]. The small drop in the performance of the experimental AI model as a result of replacing the background parenchyma and the nodule with uniform density (AUC 0.915 vs 0.926) suggests that nodule internal texture carries a small amount of predictive information, somewhat analogous to the ‘nodule type’ term in the Brock model. Several recent radiomics studies have explored the potential role of internal texture in nodule classification [26].

Ablating all information on the nodule margins by implanting a sphere of equivalent volume and mean density as the nodule into the lung parenchyma produced a reasonable performance (AUC 0.889); this is analogous to Brock without spiculation (AUC 0.862). Doing this significantly lowers the performance of both AI and Brock suggesting that nodule margins carry a significant amount of information, a concept that is well recognised by radiologists. In addition, this finding is compatible with predictive features lying within the peritumoural region, which has been shown to improve the classification of malignant nodules in the field of radiomics [27].

The performance of the experimental AI model was poorer for part-solid compared to solid nodules. Interestingly, this finding was not limited to the ablation experiments where mean nodule density was employed, and was also seen with unabated images. The relatively small numbers of part-solid nodules available to train the AI may account for this. Moreover, it is possible that indolent malignant part-solid nodules may have been mis-classified as benign over the 6.5-year NLST median follow-up period [28].

Our findings have implications for future research. First, we have demonstrated that nodule morphology plays a large role in AI prediction, with background parenchyma playing a limited, but still important, role. Future work using feature ablation can further our understanding; e.g., repeating this experiment in a population including never-smokers will yield insights about the role of the background parenchyma. Second, LCP-CNN outperformed Brock supplemented by automated measurement despite being blind to the clinical factors used in Brock such as age, family history of cancer, and sex. Prior studies in different cohorts also found this [13, 14]. It was previously found that clinical variables (e.g. age, sex, and smoking history) did not contribute significantly to LCP-CNN performance; hence, clinical variables were excluded during the derivation of the model [14]. In the future, understanding the reasons for this may help reveal how much clinical information is implicitly learned from the images.

There are several limitations to this study. First, the accuracy of the Brock model in this study is somewhat lower than in the PanCan and BCCA cohorts used to develop Brock (AUC > 0.90) [8]. Similar differences have been reported in another secondary analysis of NLST data using Brock and are likely due to underlying differences between cohorts [5]. Second, the predictive value of the Brock model is contingent on the prevalence of lung cancer in the population. This may differ in a clinical cohort from the 5.5–5.6% seen in screening cohorts such as NLST, PanCan, and BCCA [8, 15]. Third, the selection criteria applied to the NLST cohort and to this analysis limit the generalisability to clinical practice. Patients outside the age of 55–75 years, with previous lung cancer, recent chest CT, haemoptysis, or unexplained weight loss were all excluded from NLST [15]. Nodules measuring < 6 mm and ground glass opacities were excluded from this analysis. Patients with incidental pulmonary nodules in clinical practice can fall outside these criteria. Fourth, the manual diameter measurements from NLST were used directly, rather than being performed again, which may have resulted in bias in the predictive performance for the manual measurement. Fifth, there is likely bias when comparing LCP-CNN and Brock in this analysis as LCP-CNN was trained using data from NLST whilst Brock was trained on a separate population. However, direct comparison of the two models is not the main aim of this paper, and would require testing in a previously unseen population. Finally, ablated CT images are atypical images that are not seen in clinical practice and that are challenging to interpret. It is difficult to attribute the effects of ablation to a single factor; e.g., translating by 15 mm in order to ablate a nodule may, in part, reduce predictive accuracy because the local severity of emphysema is altered. Future work performing feature removal using different techniques is necessary in order to draw stronger conclusions.

Lung nodule risk prediction models lie on a continuum from the fully manual LR Brock model, to using automated segmentation to supplement this, to the fully automatic LCP-CNN which does not require nodule measurement or data entry. The performance of the Brock model improved with automated measurement, although not to the level of the LCP-CNN suggesting the latter may utilise features outside of Brock for prediction. Following feature ablation, we found that nodule size and morphology play the largest role in AI prediction, with nodule internal texture and background parenchyma playing a limited role. This was broadly analogous to the relative importance of morphological factors over clinical factors within the Brock model. These findings have important implications for future work on understanding AI prediction.

## Supplementary Information

ESM 1 (DOCX 317 kb)

## Abbreviations

AI: Artificial intelligence
AI-IA: Artificial intelligence information-ablated
AUC: Area under the receiver operating characteristic curve
BCCA: British Columbia Cancer Agency
BTS: British Thoracic Society
CI: Confidence intervals
CT: Computed tomography
LCP-CNN: Lung Cancer Prediction convolutional neural network
NLST: National Lung Screening Trial
PACS: Picture archiving and communication system
PanCan: Pan-Canadian Early Detection of Lung Cancer Study

## Declarations

### Guarantor

The scientific guarantor of this publication is Professor Fergus V Gleeson.

### Statistics and biometry

One of the authors (ND) has significant statistical expertise.

### Study subjects or cohorts overlap

Some study subjects or cohorts have been previously reported in brief in two conference proceedings at Radiological Society of North America (RSNA) and International Association for the Study of Lung Cancer (IASLC). Both conference proceedings have been included in full within this submission marked as ‘Not for Review’.

The conference proceedings reported preliminary work that focused on the adaptation of the U-net deep-learning segmentation algorithm for the automated calculation of diameter using the NLST dataset. The current study significantly expands on this. The current study evaluates the predictive accuracy of the model Brock using automated and manual diameter in comparison to an AI algorithm that does not require diameter measurement, and explores the predictive factors used by the AI model using feature removal in a manner analogous to the covariates used within the Brock model. In addition, the current work studies a more clinically relevant population, by excluding participants with nodules < 6 mm, which do not warrant follow-up, and masses > 30 mm. The authors believe that the greater scope of the current study and its findings are of interest to the readers of *European Radiology*.

We declare that this manuscript is original, has not been published before, and is not currently being considered for publication elsewhere.

### Methodology

• retrospective

• diagnostic or prognostic study

• performed at one institution

## References

[1] Iniguez CB, Kwon N, Jacobson F (2018). Estimating incidence of solitary pulmonary nodules: novel methods using claims data to answer unknown epidemiological questions. Chest.

[2] Baldwin DR, Callister MEJ (2015). The British Thoracic Society guidelines on the investigation and management of pulmonary nodules. Thorax.

[3] MacMahon H, Naidich DP, Goo JM (2017). Guidelines for management of incidental pulmonary nodules detected on CT images: from the Fleischner Society 2017. Radiology.

[4] White CS, Dharaiya E, Campbell E, Boroczky L (2017). The Vancouver Lung Cancer Risk Prediction Model: assessment by using a subset of the National Lung Screening Trial Cohort. Radiology.

[5] Nair VS, Sundaram V, Desai M, Gould MK (2018). Accuracy of models to identify lung nodule cancer risk in the National Lung Screening Trial. Am J Respir Crit Care Med.

[6] Winter A, Aberle DR, Hsu W (2019). External validation and recalibration of the Brock model to predict probability of cancer in pulmonary nodules using NLST data. Thorax.

[7] Al-Ameri A, Malhotra P, Thygesen H (2015). Risk of malignancy in pulmonary nodules: a validation study of four prediction models. Lung Cancer.

[8] McWilliams A, Tammemagi MC, Mayo JR (2013). Probability of cancer in pulmonary nodules detected on first screening CT. N Engl J Med.

[9] Nair A, Bartlett EC, Walsh SLF (2018). Variable radiological lung nodule evaluation leads to divergent management recommendations. Eur Respir J.

[10] Revel MP, Bissery A, Bienvenu M (2004). Are two-dimensional CT measurements of small noncalcified pulmonary nodules reliable?. Radiology.

[11] van Riel SJ, Ciompi F, Jacobs C (2017). Malignancy risk estimation of screen-detected nodules at baseline CT: comparison of the PanCan model, Lung-RADS and NCCN guidelines. Eur Radiol.

[12] Tammemagi M, Ritchie AJ, Atkar-Khattra S (2019). Predicting malignancy risk of screen-detected lung nodules–mean diameter or volume. J Thorac Oncol.

[13] Baldwin DR, Gustafson J, Pickup L (2020). External validation of a convolutional neural network artificial intelligence tool to predict malignancy in pulmonary nodules. Thorax.

[14] Massion PP, Antic S, Ather S et al (2020) Assessing the accuracy of a deep learning method to risk stratify indeterminate pulmonary nodules. Am J Respir Crit Care Med. 10.1164/rccm.201903-0505oc 10.1164/rccm.201903-0505OC PMC7365375 32326730

[15] Aberle DR, Adams AM, Berg CD (2011). Reduced lung-cancer mortality with low-dose computed tomographic screening. N Engl J Med.

[16] Ronneberger O, Fischer P, Brox T (2015) U-net: convolutional networks for biomedical image segmentation. In: Lecture Notes in Computer Science (including subseries Lecture Notes in Artificial Intelligence and Lecture Notes in Bioinformatics). Springer Verlag, pp 234–241

[17] Calculator: Solitary pulmonary nodule malignancy risk in adults (Brock University cancer prediction equation) - UpToDate. https://www.uptodate.com/contents/calculator-solitary-pulmonary-nodule-malignancy-risk-in-adults-brock-university-cancer-prediction-equation. Accessed 3 Apr 2020

[18] Siddique N, Sidike P, Elkin C, Devabhaktuni V (2020) U-Net and its variants for medical image segmentation: theory and applications. IEEE Access. 10.1109/ACCESS.2021.3086020

[19] Bankier AA, MacMahon H, Goo JM (2017). Recommendations for measuring pulmonary nodules at CT: a statement from the Fleischner Society. Radiology.

[20] Arteta C, Dowson N, Novotny P (2018). MA20.11 automatic nodule size measurements can improve prediction accuracy within a Brock risk model. J Thorac Oncol.

[21] Ather S, Arteta C, Dowson N et al (2018) Nodule size measurement: automatic or human-which is better for predicting lung cancer in a Brock model? In: Rsna 2018. http://archive.rsna.org/2018/18022203.html. Accessed 4 Apr 2020

[22] Chihara L, Hesterberg T (2018) Mathematical statistics with resampling and R, 2nd edition | Wiley, 2nd ed. Wiley

[23] Devaraj A, van Ginneken B, Nair A, Baldwin D (2017). Use of volumetry for lung nodule management: theory and practice. Radiology.

[24] Tang LYW, Coxson HO, Lam S (2020). Towards large-scale case-finding: training and validation of residual networks for detection of chronic obstructive pulmonary disease using low-dose CT. Lancet Digit Heal.

[25] Pickup L, Declerck J, Munden R (2017). MA 14.13 nodule size isn’t everything: imaging features other than size contribute to AI based risk stratification of solid nodules. J Thorac Oncol.

[26] Khawaja A, Bartholmai BJ, Rajagopalan S (2020). Do we need to see to believe?—radiomics for lung nodule classification and lung cancer risk stratification. J Thorac Dis.

[27] Beig N, Khorrami M, Alilou M (2019). Perinodular and intranodular radiomic features on lung ct images distinguish adenocarcinomas from granulomas. Radiology.

[28] Hammer MM, Palazzo LL, Kong CY, Hunsaker AR (2019). Cancer risk in subsolid nodules in the National Lung Screening Trial. Radiology.

