# Supplementary material for: Developing an understanding of artificial intelligence lung nodule risk prediction using insights from the Brock model
Source: Eur Radiol. 2022 Mar 3;32(8):5330–8. doi: 10.1007/s00330-022-08635-4 (PMC9279235; doi:10.1007/s00330-022-08635-4)

Supplementary Table 1: Patient demographics and characteristics of pulmonary nodules for used for training the U-Net nodule segmentation algorithm. All patients had benign nodules in order to preserve the malignant nodules for the main analysis.

| Participants (n, %) | 1276 (100.0) |
| --- | --- |
| Age (years) (mean, SD) | 62.0 (5.2) |
| Female (n, %) | 569 (44.6) |
| Current smoker at enrolment (n, %) | 632 (49.5) |
| Smoking history (pack-years) (mean, SD) | 55.6 (22.5) |
| Years since quitting smoking (mean, SD) | 7.2 (4.7) |
| Emphysema (by study) (n, %) | 457 (35.8) |
| Personal history of cancer (n, %) | 51 (4.0) |
| Family history of cancer (n, %) | 277 (21.7) |
|  | |
| Pulmonary nodules (n, %) | 3096 (100.0) |
| Solitary nodule (n, %) | 630 (49.4) |
| Upper lobe location (n, %) | 951 (30.7) |
| Spiculation (n, %) | 192 (6.2)) |
| Part-solid nodule (n, %) | 100 (3.2) |

Abbreviations: n, number; %, percentage; SD, standard deviation.

Supplementary Figure 1: Diameter distribution for benign and cancerous nodules using manual diameter measurement, automatic axial diameter and automatic equivalent spherical diameter.


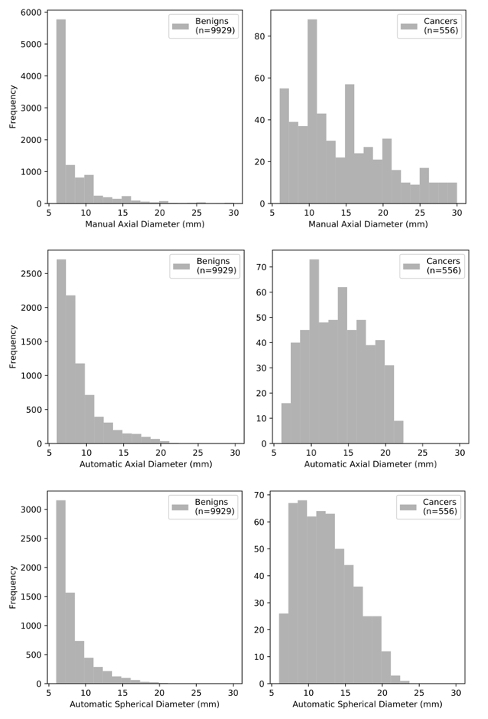


Supplementary Figure 2: Distribution of Brock risk of malignancy score for benign and cancerous nodules within the feature-reduced Brock models. For display purposes, modified Brock models were renormalised so that the mean modified score is 50.


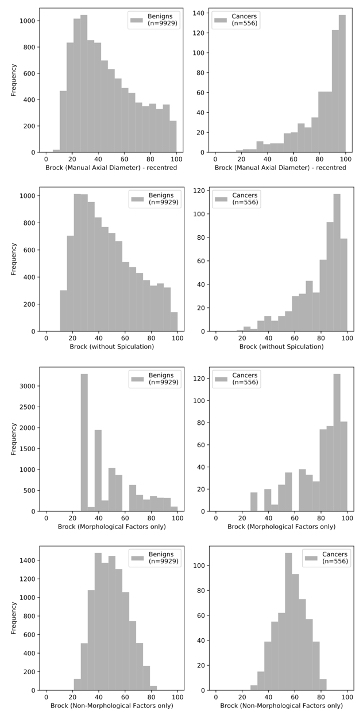


Supplementary Figure 3: Distribution of artificial intelligence (AI) risk of malignancy score for benign and malignant nodules within the information-ablated AI models.


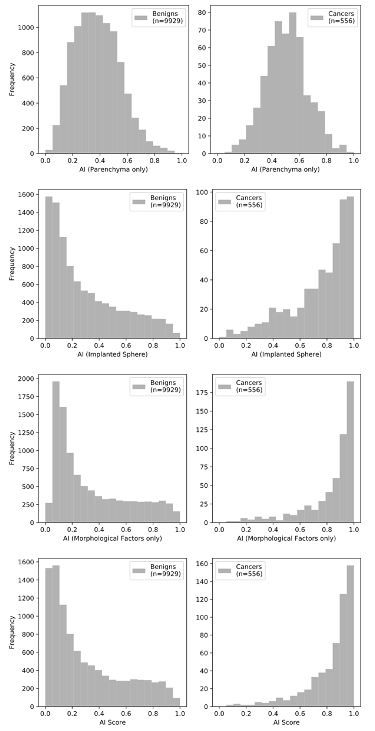

Supplement: Supplementary file 1 — (DOCX 317 kb) [file 330_2022_8635_MOESM1_ESM.docx]
